# Collectanea

**Published:** 1827-12

**Authors:** 


### COLLECTANEA.

Floriferis ut apes in saltibus omnia libant,
Omnia nos, itidem, depascimur aurea dicta.

### PHYSIOLOGY.

Mr. Rumball *on the Focus of Vision.*—I affirm that " the images of objects are *not* inverted upon the retina, but that every image is painted there in a point;" in other words, " *the focus of the eye is upon, and not anterior to, the retina.*"

Exp. 1.—Dissect off the coats of the eye at the posterior extremity of its axis. Hold it up between the finger and thumb, and the vitreous humor will protrude. Look through the eye, and pass a probe or other object backwards and forwards before the pupil. *Its apparent motion will be the reverse of its real one.*

Exp. 2.—Cut away the protruding vitreous humor. Now pass a probe up and down before the pupil; and, upon looking through the eye, the probe's real and apparent motion will be the same.

In the first experiment, the axis of the eye is slightly elongated; and, as the image of the probe is inverted, we have sufficient proof that the rays of light proceeding from it must have crossed each other, before their exit from the eye. *The focus is therefore within the axis.*

In the second experiment, the axis is shortened, and the real and apparent position of the object being the same, demonstrates that the rays have not crossed; consequently the focus of the eye is now without, or posterior to it. But, as the retina is situate between the point where the first experiment proves the rays to have crossed, and the point which the second experiment determines to be anterior to their crossing; and, as their extreme points approximate closely to each other, as in the first case the focus is within, and in the second without, the axis, and as the retina is situate between them, I consider my proposition established, viz. " *that the focus of the eye is upon the retina.*" (*Philosophical Magazine.*)

---

*Experiments on the Functions of the Ear.*—The following details are taken from a communication, by Mr. Wheatstone, to the Journal of Science.

§ 1. If the hand be placed so as to cover the ear, or if the entrance of the meatus auditorius be closed by the finger without pressure, the perception of external sounds will be considerably diminished, but the sounds of the voice produced internally will be greatly augmented: the pronunciation of those vowels in which the cavity of the mouth is most closed, as *e*, *o*, *u*, &c. produce the strongest effect: on articulating smartly the syllables *te* and *kew*, the sound will be perfectly loud.

Placing the conducting stem of a sounding tuning-fork* on any part of the

* The tuning-fork consists of a four-sided metallic rod, bent so as to form two equal and parallel branches, having a stem connected with the lower curved part of the rod, and contained within the plane of the two branches. The branches are caused to vibrate by striking one end against a hard body, whilst the stem is held in the hand. The sound produced by this instrument, when head, when the ears are closed as above described, a similar augmentation of sound will be observed. When one ear remains open, the sound will always be referred to the closed ear; but, when both ears are closed, the sound will appear louder in that ear the nearer to which it is produced. If, therefore, the tuning-fork be applied above the temporal bone near either ear, it will be apparently heard by that ear to which it is adjacent; but, on removing the hand from this ear, (although the fork remains in the same situation,) the sound will appear to be referred immediately to the opposite ear.

In the case of the vocal articulations, the augmentation is accompanied by a reedy sound, occasioned by the strong agitations of the tympanum. When the air in the meatus is compressed against this membrane by pressing the hand close to the ear, or when the eustachian tube is exhausted by the means indicated by Dr. Wollaston, the reedy sound is no longer heard, and the augmentation is considerably diminished. The ringing noise which simultaneous accompanies a very intense sound, proceeds from the same cause, and may be prevented by the same means. This ringing may be produced by applying the stem of a sounding tuning-fork to the hand when covering the ear, or by whistling when a hearing-trumpet is placed to the ear. As a proof that the resulting augmentation, which, when great, excites the vibrations of the tympanum, is owing to the reciprocation of the vibrations by the air contained within the closed cavity, it may be mentioned that, when the entrance of the meatus is closed by a fibrous substance, as wool, &c. no increase is obtained.

If the meatus and the concha of one ear be filled with water, the sounds above mentioned will be referred to the cavity containing the water, in the same way as when it contained air, and was closed by the hand: it will be indifferent whether any partition be interposed between the cavity and the external air, as the water is equally well insulated by a surface of air as by a solid body.

§ 2. The preceding experiments have shown that sounds immediately communicated to the closed meatus externus are very greatly augmented; and it is an obvious inference, that if external sounds can be communicated so as to act on the cavity in a similar manner, they must receive a corresponding augmentation. The great intensity with which sound is transmitted by solid rods, at the same time that its diffusion is prevented, affords a ready means of effecting this purpose, and of constructing an instrument, which, from its rendering audible the weakest sounds, may with propriety be named a Microphone.

Procure two flat pieces of plated metal, each sufficiently large to cover the external ear, to the form also of which they may be adapted; on the outside of each plate, directly opposite the meatus, rivet a rod of iron or brass wire, about sixteen inches in length, and one-eighth of an inch in diameter, and fasten the two rods together at their unfixed extremities, so as to meet in a single point. The rods must be so curved, that, when the plates are applied to the ears, each rod may at one end be perpendicularly inserted into its corinsulated, is very weak, and can only be distinctly heard when its branches are brought close to the ear; but instantly its stem is connected with any surface capable of vibrating, a great augmentation of sound ensues from the communicated vibrations. The facility of its insulation and communication renders it a very convenient instrument for a variety of acoustical experiments.

responding plate, and at the other end may meet before the head in the plane of the mesial line. The spring of the rods will be sufficient to fix the plates to the ears; but for greater security ribands may be attached to each rod near its insertion in the plate, and be tied behind the head.

A more simple instrument may be constructed to be applied to one ear only, by inserting a straight red perpendicularly into a similar plate to those described above.

The Microphone is calculated only for hearing sounds when it is in immediate contact with sonorous bodies: when they are diffused by their transmission through the air, this instrument will not afford the slightest assistance.

It is not my intention in this place to detail all the various experiments which may be made with this instrument; a few will suffice to enable the experimenter to vary them at his pleasure.

1. If a bell be rung in a vessel of water, and the point of the microphone be placed in the water at different distances from the bell, the differences of intensity will be very sensible. 2. If the point of the microphone be applied to the sides of a vessel containing a boiling liquid, or if it be placed in the liquid itself, the various sounds which are rendered, may be heard very distinctly. 3. The instrument affords a means of ascertaining, with considerable accuracy, the points of a sonorous body at which the intensity of vibration is the greatest or least: thus, placing its point on different parts of the sounding-board of a violin or guitar, whilst one of its strings is in vibration, the points of greatest and least vibration are easily distinguished. 4. If the stem of a sounding tuning-fork be brought in contact with any part of the microphone, and at the same time a musical sound be produced by the voice, the most uninitiated ear will be able to perceive the consonance or dissonance of the two sounds; the roughness of discords, and the beatings of imperfect consonances, are thereby rendered so extremely disagreeable, and form so evident a contrast to the agreeable harmony and smoothness of two perfectly consonant sounds, that it is impossible that they can be confounded.

§ 3. Apply the broad sides of two sounding tuning-forks, both being unisons, to the same ear; on removing one fork to the opposite ear, allowing the other to remain, the sensation will be considerably augmented.

It is well known, that when two consonant sounds are heard together, a third sound results from the coincidences of their vibrations; and that this third sound, which is called the grave harmonic, is always equal to unity, when the two primitive sounds are represented by the lowest integral numbers. This being premised, select two tuning-forks, the sounds of which differ by any consonant interval excepting the octave; place the broad sides of their branches, while in vibration, close to one ear, in such a manner that they shall nearly touch at the acoustic axis, the resulting grave harmonic will then be strongly audible, combined with the two other sounds; place afterwards one fork to one ear, and the consonance will be heard much richer in volume, but no audible indications whatever of the third sound will be perceived.

§ 4. Very acute sounds, such as the chirping of the gryllus campestris, &c. are rendered inaudible by exhausting the air from the eustachian tube, and thereby producing a tension of the membrane of the tympanum: the different thicknesses or tensions of this membrane may therefore occasion that diversity of the limits of audibility, with regard to the acute sounds which Dr. Wollaston has pointed out as existing in different individuals; if so, it would be desirable to ascertain this limit in individuals in whom the tympanum is perforated or destroyed.

§ 5. When the auricula is brought forward, all acute sounds are rendered much more intense, but no sensible difference is perceived with regard to the grave sounds. The higher tones of glass staccados, or of an octave flute, the ticking of a watch, all kinds of sibilant sounds, &c. are thus greatly augmented: the experiment is easily tried, by whistling very shrill notes. A still greater augmentation of the acute sounds is obtained, by placing the hands formed into a concave behind the ears, and by bending downwards the upper part of the auricula, so as to obtain a more complete cavity.

§ 6. I will conclude with the following observation:—I had, in consequence of a cold, a very slight pain in my left ear; on sounding the regular notes of the piano-forte, $C^3$ and $C^4$ were much louder than the others, and the loudness was much increased by placing the hand in the manner above described to the left ear. When it was pressed close, or when the eustachian tube was closed, the intensities of all the notes were equalised. I attribute this affection to the diminished tension of the membrana tympani, which was again increased by the operation described.

---

*Fascination of Snakes.*—I have (says Mr. Nash) often heard stories about the power that snakes have to charm birds and animals, which, to say the least, I always treated with the coldness of scepticism; nor could I believe them until convinced by ocular demonstration. A case occurred in Williamsburgh (Mass.), one mile south of the house of public worship, by the way side, in July last. As I was walking in the road at noon-day, my attention was drawn to the fence by the fluttering and hopping of a robin red-breast, and of a cat-bird, which upon my approach flew up, and perched on a sapling two or three rods distant; at this instant a large black snake reared his head from the ground near the fence. I immediately stepped back a little, and sat down upon an eminence. The snake in a few moments slunk again to the earth, with a calm placid appearance, and the birds soon after returned, and lighted upon the ground near the snake; first stretching their wings upon the ground, and spreading their tails, they commenced fluttering round the snake, drawing nearer at almost every step, until they stepped near or across the snake, which would often move a little or throw himself into a different posture, apparently to seize his prey; which movements, I noticed, seemed to frighten the birds, and they would veer off a few feet, but return again as soon as the snake was motionless. All that was wanting for the snake to secure his victims seemed to be, that the birds should pass near his head, which they would probably have soon done, but at this moment a waggon drove up, and stopped. This frightened the snake, and it crawled across the fence into the grass: notwithstanding, the birds flew over the fence into the grass also, and appeared to be bewitched to flutter around their charmer, and it was not until an attempt was made to kill the snake, that the birds would avail themselves of their wings, and fly to a forest one hundred rods distant.

The movements of the birds while around the snake seemed to be voluntary, and without the least constraint; nor did they utter any distressing cries, or appear enraged, as I often have seen them when squirrels, hawks, and mischievous boys attempted to rob their nests, or to catch their young ones; but they seemed to be drawn by some allurement or enticement, and not by any constraining or provoking power: indeed, I thoroughly searched all the fences and trees in the vicinity to find some nest or young birds, but could find none.

What this fascinating power is, whether it be the look, or effluvium, or the singing by the vibrations of the tail of the snake, or any thing else, I will not attempt to determine; possibly this power may be owing to different causes in different kinds of snakes. But so far as the black snake is concerned, it seems to be nothing more than an enticement or allurement with which the snake is endowed to procure his food.

The Editor adds:—In the month of June, 1823, in company with a friend, I just crossed the Hudson river, from the town of Catskill, and was proceeding in a carriage, by the river, along the road, which is here very narrow, with the water on one side and a steep bank covered with bushes on the other. Our attention was in this place arrested by a number of small birds, of different species flying across the road and then back again, and turning and wheeling in manifold gyrations, and with much chirping, yet making no progress from the particular place over which they fluttered. We were not left long in doubt, when we observed a black snake of considerable size, partly coiled and partly erect from the ground, with the appearance of great animation, his eyes brilliant, and his tongue rapidly and incessantly brandished. This reptile, we perceived to be the cause and the centre of the wild motions of the birds, which ceased as soon as the snake, alarmed by the approach of the carriage, retired into the bushes; the birds, however, alighted upon the neighbouring branches, probably awaiting the re-appearance of their tormentor and enemy. Our engagements did not permit us to wait to see the issue of this affair, which seems to have been similar to that observed by Mr. Nash.—SILLIMAN's *Journal.*

---

*Experiments on Tendons.*—It will be recollected that some time since we mentioned a case of rupture of the tendo-achilles, successfully treated by Dr. Horner with a seton; this case, although sufficient to attest its efficacy, required further testimony to establish its application to even analogous cases with perfect confidence; with this view Dr. F. made the following experiment on a dog.

July 21st, 1826.—A small puncture was made through the integuments, and the posterior third of the tendo-achilles divided an inch and a quarter above its insertion. The dog was permitted to run loose, and in a few hours the whole of the tendon was found completely divided, and the ends loose and separated to a considerable distance.

No attention was paid to the dog until August 24th; the inflammation had completely subsided, the ends of the divided tendon were separated some distance. Friction was employed to induce inflammation, and by means of bandage and splint, a proper position of the parts was maintained for sixteen days, when, no benefit resulting, the dog was again liberated. October 21st.—No difference in the situation of the parts, a needle, armed with a small silk riband was passed between the retracted ends of the tendon, and the animal set at liberty. 23d.—Considerable inflammation induced by the seton, dressings applied to keep the ends of the tendon as nearly as possible in a proper position, and the dog at rest; suppuration freely established at the end of twelve days, when the seton, bandages, &c. were removed. November 21st.—Cure perfect; the dog uses each leg with equal facility. The tendon uniformly enlarged, from the muscle to the os calcis. The dog was now killed, unnecessary cruelty and, on dissection, the tendon, which was re-united, was found to extend "uniformly from its origin to its insertion, no enlargements or depressions being perceptible, where the ends of the old tendon were united to the ends of the newly-formed substance. The whole cord was rounder, and harder, than its fellow; not so flexible, and not possessing that beautiful pearly lustre so conspicuous in a healthy tendon."

In order to illustrate the condition of parts, at the time the seton was employed, the tendo-achilles of another dog was partially divided; the tendon was then ruptured, as in the preceding case. At the end of a month, the dog was killed, and dissection presented no traces of inflammation. "The absorbents had rounded the ends of the tendon, which were separated two inches from each other. No evidences were here given of that slow process instituted by nature, of which some authors have spoken." The result of these experiments we consider perfectly conclusive, in support of the utility of the seton.

Some experiments were next instituted, with a view of testing the value of the ancient practice in cases of ruptured tendons, by sewing their ends together. In one instance, the tendon tore itself from the ligature, which remaining in the wound, acted as a seton; in another, by the ligature, the ends of the tendon were retained nearly in contact, and direct union took place. In some instances before dissection, the limb was injected with fine size, in order to display the vascularity of the newly-formed parts.

The process by which re-union is produced is worthy of attention. "The sheathe of the tendon and the cellular substance in the vicinity assume the inflammatory action with much more facility than the tendon itself. They therefore arrive at a height of action adequate to the effusion of lymph sooner than the extremities of the tendon, and consequently furnish the first bond of union. Into this lymph vessels shoot from all the adjacent parts capable of affording them, and it soon becomes an organized mass. The ends of the tendon are so slow in developing their vessels, that they are not able to furnish their quota to the coagulated lymph, and are therefore not permitted to connect themselves with the newly-formed substance. The ends of the tendon were found, in several of the experiments, loose, changed in colour, and surrounded by a dark sanious fluid, while the newly-formed medium of connection was attached a short distance from the end to the tendon laterally. If the envelopes of an artery be too much disturbed, and the vasa vasorum injured, the artery dies, to where they are furnished in sufficient quantities for its health; in the same way, if the sheathe of the tendon furnishing the vessels essential to its vitality be disturbed by rupture, or other means, it must die. In process of time, these dead ends of the tendon, sanious blood, &c. are removed by the absorbents, and the whole cord becomes a continuous line, of nearly the same diameter from the muscles to its insertion. The primary inflammation seldom or never surpasses the adhesive point, unless the neighbouring parts are also much injured. From a want of vascularity in the parts in a natural state, we have here a low grade of inflammation, not to be restrained by purging, rest, low diet, &c. as recommended by most authors, but which requires the opposite course of treatment."—*Philadelphia Journal.*

### PATHOLOGY.

*Cases of false Consecutive Aneurism of the Heart, and true Aneurism of the Arteries. By* G. BRESCHET, M. D., &c. &c.—*Repertoire d'Anatomie, tome 3, No. II.* 1827.—False consecutive aneurism of the heart is a disease much less known than other maladies to which that organ is liable. In the general description of the diseases of the circulating system, it is remarkable that this species of aneurism of the heart has been considered never to occur. Authors have confined themselves to the history of cases in which there existed a dilatation of the ventricles or auricles, with either an increase or diminution of the parietes; but aneurisms of the heart, accompanied by rupture, have not been mentioned. The reverse is the fact with respect to the arteries. Most pathologists have disputed the simple dilatation of these vessels without rupture of one or more of their coats, and have not been inclined to admit the existence of true aneurism from arterial dilatation. The object of M. Breschet's memoir is to prove, by cases, the existence of both these varieties of aneurism. A plate is given of the appearances detected on dissection in the heart of the celebrated Talma. At the apex of the heart was found a considerable enlargement, which was lined by the pericardium. The left ventricle was dilated : its parieties had suffered no perceptible alteration. The cavity of the ventricle was sufficiently enlarged to admit a hen's egg of an ordinary size. At the inferior part of the ventricle there was a communication, with a round pouch, by means of an opening about an inch in diameter. This communication between the ventrical and aneurismal pouch was established by means of a thick cartilaginous band (*virole.*) This band gradually became thinner, and terminated in a membrane which mingled with the laminæ of the pericardium, and constituted the aneurismal pouch. It was ascertained upon a further examination that the internal membrane of the heart was ruptured. In fact, false consecutive aneurism of the heart existed. In the ten cases introduced into this memoir, there is one part which merits attention. Most of the aneurisms were found at the upper part of the left ventricle. M. Breschet believes that no such appearance has ever been detected in the right ventricle; and he infers, therefore, that the disease depends upon a purely mechanical cause. The upper part of the left ventricle has to maintain the powerful contractile efforts of the heart, and is much more likely to yield than the right, which, equal in strength, has much less violent action to support. The difficulty of determining the manner in which the disease is originally formed is acknowledged. The progress of false consecutive aneurism of the heart is sow and gradual. It would appear that, in some cases, many years have elapsed between the commencement and perfect formation of the disease. The above is but a brief abstract of M. Breschet's paper, from which the following general conclusions may be deduced :—

1. That false consecutive aneurism of the heart is generally situated in the left ventricle.
2. That the apex of the heart is the most ordinary seat of the disease.
3. That this affection is certainly an aneurismal tumor.
4. That this aneurism, from the mode of its formation, the state of its parieties, and the contents of the cyst, closely resembles false consecutive aneurism of the arteries in the sense given by Scarpa to the term aneurism.
5. That the heart may be affected with the same disease as that with which the arteries are most commonly attacked, and that thus there is established between the diseases of the heart and arteries an analogy, which might be anticipated from the anatomy of their structure, their original formation, and subsequent development.

---

*Erysipelas.*—Dr. De Leon gives an account of an epidemic that prevailed during the early part of 1826, in Kingston, Jamaica, West Indies. Early in December, 1825, and through the following month, several distinct cases of erysipelas presented themselves, and about the middle of May, 1826, distinct inflammati on of the cellular membrane appeared in one or two persons, while in by far the majority, erysipelas and inflammation of the cellular membrane occurred simultaneously. After a short time many persons were affected with cough, and Dr. De L. expresses his belief that what appeared influenza, was a modified form of the erysipelatous epidemic, from the fact that many thus affected at length became the subjects of erysipelas, in one or other of its multiform appearances. In some cases the attack was sudden, "one victim to the disease was awakened out of her sleep with severe shivering, to which in less than an hour succeeded all the symptoms of the disease in a highly-aggravated degree; and at the expiration of three days she was a corpse." In the majority of cases, however, the disease was of slow growth; the patient complaining of the usual precursors of an attack of fever, gradually increasing in severity, when the throat became affected with a sense of fulness and tickling, unaccompanied with soreness or pain. "On looking into the throat, the appearances observed were singular and remarkable, so that it was impossible to refer them to any disease described by nosologists. A darkly-coloured purplish inflammation commenced from the curtain of the palate, spread over the uvula and over the tonsils, and became, in some cases, fainter and fainter as it descended over the pharynx; while in others the pharynx, as far down as could be seen, was equally inflamed and purplish. Sometimes several veins were seen on the pharynx turgid with dark blood. The tonsils were almost always more or less swollen, and in severe cases covered with small ulcers, which were generally charged with a whitish yellow excretion, and were of a circular figure. They often attacked the uvula and the pharynx, while the mucous membrane became puffy and purplish." In some instances, the papillæ of the tongue were swollen and raised, "so that it appeared sprinkled with red acuminated elevations." The external glands of the neck, axilla, and groin, were frequently inflamed and swollen.

"In one case, the cellular texture about the arm and back, and the cellular texture and skin of one of the nates, were simultaneously attacked with inflammation. Suppuration took place in the nates, and large quantities of matter were daily discharged;" in another, the whole surface of the abdomen was attacked with a deep-coloured redness, which vanished on pressure and gave no pain, it spread rapidly down the thighs, on which diffused tumors formed, "very painful, hard, massy, and dark red, and terminated in suppuration, and the discharge of a great quantity of matter, with shreds of ruptured cellular membrane." These tumors, also the enlarged glands, were sometimes dissipated by the immediate and repeated application of leeches and hot linseed-meal poultices. The disease, when properly treated, was rarely fatal.

*Treatment.*—In cases characterized by high excitement, the saline draught, with liq. ammon. acet. and sp. æther, sulph., and purgatives so as to produce several evacuations in the twenty-four hours, were advantageous. When an abatement of the high pyrexial symptoms took place, liquor arsenicalis, with saline mixture and infusion of quassia, and sulphate of quinine, were administered. In debilitated subjects the two best remedies were camphor and the carb. ammon. To allay the irritability of the stomach, tinct. opii, with the effervescing mixture. Leeches over the epigastrium, when the disease was of an ardent character, followed by a blister, were highly serviceable; and, when the vital energies were in a sinking condition, pulv. cinnamomi comp. and confectio aromatica, with the camphor and ammonia, and spiced brandy with the nourishment.

"Blisters over the chest diminished the sense of constriction and the difficulty of breathing, which were sometimes present in hysterical subjects. Inhalation of sulphuric æther, or of the aromatic spirits of ammonia, alleviated the anxious sense of suffocation, frequently the most imminently alarming symptom by which the patient was harassed. Gargles of the stimulant and astringent kind were most useful." The ulcers within reach were touched with corrosive sublimate, or with nitrate of mercury in solution. Mercury was only prescribed when the function of the liver appeared to be disturbed. —*New York Medical and Physical Journal.*

---

*Test of Sound Lungs.*—Dr. Lyons, (staff surgeon,) in a letter to the editors of the *Edinburgh Medical and Surgical Journal,* says "The plan which I principally confide in for the purpose of ascertaining the integrity of the lungs of recruits, since the commencement of this year, continues to answer my most sanguine expectations; and, as it appears to meet your approbation, as well as that of many of my Edinburgh medical friends, I beg that you will obtain for it a place in the Medical Journal, in order that it may become more extensively known.

Most officers placed in a situation similar to myself will speedily recognize in it a much more applicable assistant in difficult chest cases than the stethoscope; for by the simple process in which this plan consists they can ascertain at once whether the respiratory organ labours under any diminution of its capacity by the formation of vomicæ, or from the deposition or infiltration of tubercular matter, coagulable lymph, hepatization, or matter between the pleura costalis and pulmonalis; it likewise detects any morbid irritability of the bronchial tubes, which give rise to dyspnœa. When the deaths from phthisis among the troops, and the numerous cases of young and well-looking phthisical subjects, who daily present themselves for admission into the service, are considered, and the difficulties which the most experienced medical officers often meet with in ascertaining precisely the state of their lungs, it must be allowed that any plan which would obviate the difficulty would prove highly useful to the military and naval service. Most of my professional friends to whom I have mentioned the plan are of opinion, that, in exploring the chest in the incipient states of tubercular phthisis, and in pleuritis with effusion, it cannot fail to prove of great utility to medical men. In the latter of these diseases it readily detects the progress of absorption of the fluid, and is not liable to the objections which are raised against the stethoscope, on the grounds of delicacy, or exposure to the surrounding atmosphere. In many instances the diagnoses formed from this plan were corroborated by the stethoscope, percussion, or immediate auscultation, as well as by the confession of the individuals, after their hopes of entering the service were destroyed.

The patient, after being examined for external causes of disqualification, is desired to inspire fully, and during the following expiration to count as far as he can slowly and audibly, without allowing himself to take another inspiration. The number of seconds he is enabled to continue counting in the expiration is noted by a watch with a second hand: this time will be found to increase in proportion to the integrity of the lungs. In confirmed phthisis, the time of counting never exceeds eight, frequently falls under six or seven seconds; in pleuritis and pneumonia it seldom lasts for more than nine, and frequently falls as low as six and four; in hepatization it ranges, in proportion to the extent of disease, from eight to fourteen. In a young man, strong-looking and healthy, discovered to have dyspnœa, with a slight tinge of lip and ear, and also in an asthmatic patient during the absence of the fit, although a healthy-looking young man, it did not exceed ten. In a perfect inspiration, with apparently sound lungs, the individual counts from twenty-five to thirty seconds during the expiration. After the most perfect inspiration, the time of counting during the expiration does not exceed thirty-five seconds.

A very few trials will enable the most inexperienced persons to apply this plan, and to make the necessary allowances in particular cases.

---

### PRACTICAL MEDICINE.

*Malaria.*—The following observations are extracted from the last Number of the Journal of Science:—

It is notorious that, in the last autumn, the remittent fevers in various parts of the country amounted to a species of pestilence, such as has scarcely been known in England from this cause, or we might almost indeed say, from any other disease since the days of Sydenham. Wherever ague had ever existed, or even been supposed possible, in those places was this fever found; so that, in all the well-known tracts in Lincolnshire, Norfolk, Suffolk, Kent, Essex, Sussex, Hampshire, and so forth, there was scarcely a house without one or more inhabitants under fever, while the event, as might be suspected, was a considerable mortality. In the parish of Marston, in Lincolnshire, for example, it amounted to twenty-five in 300 inhabitants; in some other places, it reached one in sixteen, one in thirteen, one in nine. And so extensive was its range, that even Hastings did not escape; while it should be almost superfluous to say that every other town on the sea-coast was so much infected by it, that they who resorted to them for bathing as usual, found themselves most awkwardly situated, and also suffered in considerable numbers.

To come nearer home, and to what must interest us of the metropolis more, the same fevers were extremely abundant in various parts of the outskirts of London, as also in the villages or towns which are connected with it, within a range of from six to ten miles. Not to enumerate all these, this was the case throughout the range of streets or houses which extends from Buckingham Gate to Chelsea; in which long line it is said that almost every house had a patient or more under this fever; though, as the author has truly observed, these were mistaken for typhus, or at least thus misnamed. Thus it was also about Vauxhall and Lambeth; and to a great extent among all that scattered mixture of town and country which follows from Whitechapel, from Bishopsgate, and so forth, and very particularly along Ratcliffe Highway, and so on, to an indefinite range along the river, not only on this side but on the opposite one, so as to include Rotherhithe, and then proceeding onward to Deptford, Greenwich, Woolwich, Plumstead, so as to carry us beyond the boundary which we proposed to notice.

And, in addition to the towns or villages which we have just named, we may enumerate Lewisham, in which we knew one house in which there were nine patients under this fever, which proved mortal to one. Dulwich, especially subject to this disorder, Fulham, Ealing, and the several other villages along the Thames as far as Chertsey; and even Richmond, where, as at Lewisham, there was one house known to us, inasmuch as being intimate friends, where ten individuals at one time were suffering under this disease.

We must not prolong this enumeration, since we might easily occupy a dozen of our pages with similar details, ranging in fact all over England; but we must still observe, that, whatever was the pestilence last year, it promises to be much greater in the present one. This is easily judged from the manner in which the season has set in; but still more decidedly from the extraordinary prevalence of ague in the spring; since that which is intermittent fever then, will be remittent in the autumn; or rather, as the author has justly remarked, there will scarcely be a definite season of vernal intermittent, but the remittent will commence immediately, increasing in extent and severity as the summer advances, and promising to become, in the autumn, the greatest season of disease that England has known for this century.

As an example of this, it must suffice to enumerate two or three facts, while these are as satisfactory for our purpose as a thousand would be. The most general of these is, that ague is at this moment extremely abundant where it was formerly so little known as not to be noticed, and that, where single cases used to occur, there are now hundreds. Thus has it prevailed at Fulham and Ealing, and in the outskirts of London, and even in the town itself; and thus does it so prevail at Greenwich, Deptford, and in the associated vicinity, that a medical friend informs us that it comprises more than two-thirds of his entire practice, which is very extensive; whereas, a few years ago, he had rarely a patient in a year. Thus, also, in the Military Hospital at Woolwich, there were in the spring 300 patients with this disease; while, in former times, we are assured that an ague was scarcely known once in five or six years.

---

### MATERIA MEDICA.

*Preservation of Cantharides.*—It is stated by M. Farines that the active part of cantharides exists only in the soft organs of the insect; that these are the parts which are attacked by a species of acarus, and that in this way the cantharides are injured. Camphor has no power of preventing the attacks of the acarus; but M. Farines believes that pyroligneous acid will be found effectual, and proposes to prepare cantharides with it, and even to kill them at the time when they are collected by submersion in it. (*Journal of Science.*)

---

*Method of increasing the Odour of Roses.*—For this purpose, according to the author of the method, a large onion is to be planted by the side of the rose-tree, in such a manner that it shall touch the foot of the latter. The roses which will be produced will have an odour much stronger and more agreeable than such as have not been thus treated, and the water distilled from those roses is equally superior to that prepared by means of ordinary rose leaves. (*Ibid.*)

### SURGERY.

*Cases of long-continued Luxation, in which severe Mischief arose from the attempt to reduce the Parts.* By M. FLAUBERT, M.D., &c. &c. (*Repertoire d'Anatomie, tome* 3, *No. I.* 1827.)—From five of the cases related by M. Flaubert, it appears that the advice given by many authors to attempt the reduction of dislocations of long standing cannot be acted upon without considerable risk. We are aware there is no novelty in this fact, but we are inclined to believe it is not unfrequently lost sight of, and that very injudicious and violent attempts are sometimes had recourse to at the risk of much injury, and with but little probability of restoring the parts to their natural position. The longer the dislocation has existed, and the greater the degree of inflammation and swelling which has succeeded the accident, the greater will be the danger of inflicting serious injury by the after attempts at reduction; for the muscles as well as the arteries are weakened, and perhaps softened, by the previous inflammation, and are hence less capable of resisting any great efforts to reduce the parts. M. Flaubert is inclined to infer, from four of the cases he has related, that paralysis of the arm, which is often attributed to luxation of the bone, is rather to be attributed to the violent attempts to reduce the dislocated joint.

---

*Strangulation of the Penis from the Socket of a Candlestick.*—A boy lately presented himself at the Hôtel-Dieu, suffering the utmost agony from retention of urine. Upon examination, the penis was found enormously swollen, of a dark red colour, and divided in the middle by a deep depression. Upon separating the parts which formed the borders of this depression, M. Dupuytren discovered a foreign metallic body, of a yellow colour. Upon further investigation, he found, to his surprise, that this substance was the socket of a candlestick, the broad end of which was towards the pelvis. The torment of the patient was excessive. He had passed no urine for three days. The bladder was greatly distended, and ascended to the umbilicus. Gangrene of the penis was already threatened, and no time was to be lost in removing the cause of all the mischief. While the instruments were being brought for the operation, the boy, who had been pressed with many questions, confessed, that having got drunk, he had taken the socket of the candlestick "*pour tout autre chose, et qu'il y avait poussé sa verge!*" that the subsequent efforts he made to withdraw the part were useless, and greatly increased his suffering. In fact, the narrow and sharp-edged opening of the socket pressed against, and of course wounded the glans penis. M. Dupuytren first, with a pair of cutting pincers, divided the broad end of the socket at two opposite points. He then, with considerable difficulty, on account of the tumefaction of the parts, separated it into two portions, by extending the incision. The penis was now easily liberated, and the urine was passed without difficulty. The patient, at the same time ashamed and delighted, immediately disappeared without even waiting to arrange his dress. The crowd through which he passed were favoured with abundant liquid proofs of the success of the operation, which had at once removed the torments he had endured from retention of urine, as well as the danger of gangrene and even death.—*Répertoire Gen. d'Anatomie.*

---

*A Pomatum-pot found in the Vagina of a Woman, who suffered herself to be treated for Cancer of the Womb.*—Ann G—, forty-five years old, presented herself at a consultation of the Hôtel Dieu, requesting assistance for a cancer of the womb, the existence of which was attested by a formal certificate, said to have been written by a physician of her province. The patient passed her urine through the urethra and vagina, and at the same time she suffered considerable pain in the vagina and in the hypogastrium. There was a constant discharge of a mixture of fetid urine, blood, mucus, and purulent matter. M. Dupuytren was surprised to feel, upon examination, a hard and rough body about two inches within the vagina. He at first conceived it was a urinary calculus formed in the vagina, in consequence of a vesico-vaginal fistula. The patient, however, confessed that the substance was a piece of stone, which had been placed there by force. Upon being questioned as to the circumstances of this unusual act of violence, she declared, that nine years ago she had been attacked upon the road to Dijon by two soldiers, who attempted to ravish her. For some time she defended herself stoutly. She was at length, however, overpowered: and they introduced a stone into the vagina! She stated that she had afterwards much pain in making water, and considerable fever. Her suffering, however, had gradually diminished; and, for the last eight years, she had experienced but little inconvenience. The same train of symptoms again occurred, and they were even more severe than at first. She was attacked with violent fever; and could only sleep by the assistance of opium. Other symptoms of general disturbance existed from the local irritation in the vagina. M. Dupuytren felt persuaded that the patient could only be relieved by the removal of the foreign body. She was placed in the same position as for the operation of lithotomy. Upon introducing a finger, M. D. discovered that the substance was a small pot, placed across the vagina. It was removed with some difficulty; and the woman now first confessed that she knew the pot was in the passage; that it was that which the soldiers had introduced, after having, with a due attention to economy, removed the pomatum it contained. It was covered with a saline incrustation, several lines in thickness. Emollient injections were subsequently employed, and the patient three days after the operation left the hospital. The fistulous opening between the bladder and vagina, which had been caused by the pressure of the foreign body, was completely cured. It was evident from the ridiculous and varying accounts given by the patient that she had introduced it herself. She was known to have laboured under an attack of nymphomania at about the time she stated the pot to have been forced into the vagina.—*Ibid.*

---

*Case of Infibulation, followed by a Schirrous Affection of the Prepuce.*—The practice of infibulation, which originated from the passion of jealousy, and which, in the ages of barbarism, was imposed by the stronger upon the weaker sex, might have been thought to be now altogether confined to the imaginary personages of La Fontaine's Tales. No trace of it can be discovered in modern times. One example, however, of the kind has occurred to M. Dupuytren; and it is remarkable that it was in the person of a man whose mistress had induced him to submit to this very-unusual mode of resisting any disposition he might have to inconstancy. Some years ago M. Dupuytren was consulted by Dr. Petroz, upon the case of M.——, the head of one of the most important manufactories in France. He was about fifty years of age, of a strong and good constitution. For a long time he had had an abundant and fetid discharge from the penis: he made water with difficulty: the prepuce was much swollen, hard, and ulcerated in different parts. So far the case presented nothing remarkable; but the curiosity of the attendants was strongly excited by observing that the prepuce had been pierced through in several places, and that the aperture and borders of these small orifices were completely covered by a perfectly-organized cutaneous tissue. M. Dupuytren determined, before he proceeded to any decisive mode of treatment, to ascertain in what manner these perforations in the prepuce had happened. The patient stated, that, when a young man, he had visited Portugal, where he had remained several years. He there formed a tender *liaison* with a young female of strong passions, and equally strong jealousy. He was devotedly attached to her, and she acquired over him the most absolute influence. One day during the transports of their mutual passion, he felt a slight pricking sensation in the prepuce; but, having his attention completely abstracted by the caresses of his fair mistress, he did not even examine from whence arose the disagreeable feeling he had experienced. But, on retiring from the embraces of the lady, he found the prepuce secured by a little golden padlock, *artistement travaillé*, of which she had kept the key! It would appear that the lady was not deficient in eloquence, for she kept her lover in good humor by her rhetoric, assisted, indeed, by occasional caresses, and persuaded him, not only to permit the padlock to remain, but to consider it a very ornamental appendage. She even gained permission to re-apply it each time, that the skin which was pierced appeared weakened; and, however incredible it may seem, she at length, "to make assurance doubly sure," put on *two* locks. M—— remained in this state for four or five years, constantly wearing one or two of the locks appended to the prepuce, the key of which was of course taken especial care of by his mistress. The consequence ultimately was that the prepuce became diseased, and a concerous affection was threatened, when M. Dupuytren was consulted. The safest and most effectual course was then adopted. The prepuce was removed by an operation nearly resembling circumcision. Under the care of M. Sanson, the cure was complete in less than three weeks. The patient has remained in perfect health.—*Ibid.*

---

### MIDWIFERY.

*Detachment of the Placenta by injection of the Funis.*—In the fourth volume of the *Journal des Progrès des Sciences Medicales*, Dr. Taroni has related a case of retention of the placenta, in which the umbilical cord was injected with water, and the separation and expulsion of the after-birth quickly followed. The case, as it is related by Dr. T. would admit of many remarks as to the propriety of the practice which was adopted, but it is our object at present merely to record the possibility of carrying this plan into effect, and to shew that, where the placenta is retained in the uterus, it may be thus separated. If cold water had not been found sufficient, a proportion of vinegar would have been added. In about two minutes after the injection of the funis, the placenta was entirely expelled. We are informed by Dr. Hopkins, that he has had recourse to this plan in two instances, with an equally-favorable result. The patients would not permit the introduction of the hand, and pressure upon the uterus, the application of cold, &c. having been tried to no purpose, about eight ounces of water were thrown up the funis with a common syringe. In two or three minutes uterine contractions took place, of which there had been previously no symptom, although two hours had elapsed after delivery, and the placenta was expelled into the vagina. Although we do not imagine that this mode of treatment can be frequently necessary in cases of retained placenta, the fact is worthy of being recorded, as it is always advantageous to increase our means of affording assistance, however seldom their employment may be required.

---

Dr. Hopkins' *Obstetric Apparatus.*—The machine contrived by Dr. H. for the purpose of illustrating the process of labour, and the separation of the placenta, reflects great credit upon his ingenuity. It is decidedly superior to any instrument of the kind we have ever seen. We could not indeed have conceived it possible that so close an imitation of the work of nature could have been effected. The parts are formed of a composition of elastic gum, and the descent of the fœtus through the pelvis is managed with such admirable adroitness, and so accurately represents the progress of labour, and the natural feel of the parts, that the student who has been accustomed to ascertain the presentation, to watch the progress of the head, and to separate the placenta upon this machine, will be completely prepared for the execution of his obstetric duties. Dr. H. contemplates some further additions and improvements, which we shall not fail to notice.

---

*Cases of violent Vomiting occurring at the commencement of Pregnancy, and appearing to depend upon a morbid state of the Uterus and Ovum. By* M. Dance, M. D., *Aide de Clinique a l'Hotel Dieu.*—At the commencement of pregnancy, females occasionally suffer from very violent and continued vomiting, which sometimes produces exhaustion and even death. The object of M. Dance is to ascertain the cause of the vomiting, and to determine the most rational mode of endeavouring to relieve it. He is inclined to believe that this very distressing symptom is less dependant upon the sympathy of the stomach with the uterus while in a healthy condition, than upon a low degree of inflammation, either of the uterus itself, or the membranes of the ovum. He would not, however, be inclined to form a decided opinion from the two cases which he has related, although they have been deemed sufficiently interesting to be recorded. In the first case the vomiting continued for three months, without any symptoms of fever, and ultimately destroyed the patient. Upon dissection, the membrana decidua was found in a state of inflammation. The stomach was perfectly healthy. In the second case, the vomiting also continued for upwards of three months, when the patient sank and died. The parieties of the uterus were found, upon examination, in a softened and diseased state. In the tissue of the womb there was also a decided engorgement of blood. The membrana decidua, also, was more vascular than common. The appearances of lesion in the stomach were but trifling. In each of these cases a variety of internal remedies and local applications were had recourse to for the purpose of arresting the vomiting, but with little or no avail. M. Dance observes, that if it were permitted to draw general conclusions from the limited evidence he has afforded, it might be inferred that the vomiting which occurs at the commencement of pregnancy, and which so obstinately continues in spite of the best-directed treatment, is to be considered as a proof of a sub-inflammatory state of the uterine system. It is suggested, therefore, that it would be more rational to oppose the continued vomiting by antiphlogistic means applied to the region of the uterus, than to direct our attention solely to the stomach, which is but secondarily affected.

It has never fallen to our lot to lose a patient in consequence of exhaustion from the almost-incessant vomiting, which not unfrequently occurs during the first months of pregnancy; but we have rarely succeeded in effectually relieving it by any treatment we have adopted. The observations of M. Dance are certainly worthy of attention.—*Repertoire d'Anatomie.*

---

*Heart Presentation.*—Mr. Tucker has related (*Medical Repository*, Nov.) the case of a fœtal monster, which led to considerable embarrassment during the delivery. After some preliminary details, he says—"I now, of course, proceeded to examine into the state of affairs, and found (to my great astonishment) that the part presenting was a large fleshy tumor, of the shape of a heart, possessing a pulsatory motion, or a regular *systole* and *diastole*. I could not help concluding that this organ was actually in the vagina. I also imagined that I could distinguish the auricles, and the larger vessels attached to the ventricles. About ten o'clock considerable hæmorrhage came on, and the *os uteri* being fully dilated, I endeavoured to push back the tumor, and to get hold of the feet, for the purpose of delivery. In doing this I had no difficulty; but, to my surprise, what I had hardly been able to believe could be *a heart presentation*, turned out to be so in reality!

"Of course, the case was one of malformation. The fœtus appeared to be full grown: and there being a division throughout the chest and abdomen, the whole contents of both cavities were exposed to view. The display of thoracic viscera arose from a separation through the median line of the sternum, which extended to the pubes; the edges of the recti muscles were reverted, as it were, and faced with a white, tendinous border. But the deviations did not end here. The right hand exhibited but one finger, and the left foot but one toe.

"The umbilical chord made its exit on the back, between the scapulæ.

"Immediately on delivery, the child cried lustily, and lived for two hours, during the greater part of which I had the heart in my hand, and witnessed the vena cava returning the blood to its right side, and its reception on the left from the pulmonary vein. The aorta and pulmonary artery were also both distinctly seen, together with their actions.

"As all the abdominal viscera were out of the cavity, by simply turning them aside I could see the descending aorta and the ascending cava."

---

### MISCELLANEOUS.

*King's Evil.*—The cure of Scrofula by the Royal Touch, is the most singular species of quackery in the history of superstition. Lord Bacon says, that imagination is next akin to miracle-working faith. There was seemingly some of both, and a little money to boot, to keep this remedy in fashion; and as each patient touched a bit of gold, we may suppose, in this, as in other complaints, that some were cured of the king's evil, who never had any other *evil* than that of poverty, which brought more fame to these royal practitioners than they deserved.

"The curing of the king's evil (says Aubrey) by the touch of the king, does much puzzle our philosophers; for whether the kings were of the house of

York or Lancaster, it did the cure; (*i. e.*) for the most part. 'Tis true, indeed, at the touching there are prayers read; but perhaps neither the king attends them, nor his chaplains."

The French kings pretend to a greater antiquity in the exercise of this miraculous gift. We do not go higher than 1066, to Edward the Confessor, but our Gallic neighbours dated their's from Clovis, 481. Mezeray says, that this king cured one of his favorites. This gift, however, seem to have fallen to decay in the time of Louis XI.; for he having an apoplexy, sent for a famous man to cure him, by the name of Francis of Poul. Francis unhappily had the evil; but alas! the saint could not cure the king; and what was equally unfortunate the king could not cure the saint.

Our King George I. had the good sense not to pretend to this marvellous power, but the French Kings kept up the farce till 1775. Louis XV. touched no less than 2000 persons, and his predecessor 2500. The kings of Scotland did not pretend to this gift, but when their James the VIth. came to the throne of England, the virtue straight appeared in him.

In 1684, John Browne, surgeon to the king, published his "Adenochoiradelogia, or king's evil swellings, together with the royal gift of healing or cure thereof, by imposition of hands, performed for above 640 years by our kings." He gives an account of the number of persons touched for the king's evil from May 1660, to Sept. 1664, by King Charles II., viz.

| | |
|---|---|
| In 1660 | 6725 |
| 1661 | 4619 |
| 1662 | 4275 |
| 1663 | 4667 |
| 1664 | 3335 |

And from another account, by Mr. Thomas Dankley, it appears, that monarch from 1667 to 1682, actually touched, on the average, 4000 people every year.

There occurred in the 30th of Charles II. (1684,) a curious trial of Thomas Rosewell, for high treason. The words in the indictment charged him with saying, that "The people made a flocking to the king, upon pretence of being healed of the king's evil, which he could not do, but that they, being priests and prophets, could by their prayers do as much." Rosewell proved it by instancing the prophet that came to Jeroboam, and reproved him at the altar of Bethel; "and the king stretched forth his arm, and did lay hold of him, and the king's hand was dried up. And the prophet prayed to the Lord, and the king's arm was restored." He was further accused of applying the story "of Dalilah and Sampson; and (says the witness) you did not question but, in the end, the ladies would serve the king as Dalilah did Sampson." Rosewell objected to the latin in the indictment; that Morbus Regni Anglici was the disease of the English kingdom, and could not be made to signify the king's evil. During the trial, Judge Jefferies told Rosewell's witnesses "We know well enough you snivelling saints can lie;" and so on. Rosewell was found guilty, but pardoned; the sole exclusive patent of curing the king's evil still lying with royalty.—WADD'S *Mems. Maxims, and Memoirs.*

---

*Specifics.*—The French government, as well as our own, has, in more than one instance, given large sums for the purchase of nostrums, with similar intentions and success.

In 1720, the French ministry purchased the "*Pulvis Carthusianum,*" or chermes mineral, originally invented by Glauber; and having been a great secret, it immediately became a great favorite with the public.

Previously to this, with the same laudable design, they purchased of Prieur de Cabrier, an arcanum, which, he boasted, would cure ruptures without bandage or operation. The recipe was then made public; when lo! the grand specific, which was to benefit all mankind, consisted of spirit of salt, with a certain quantity of red wine, *to be taken often* every day! about as great a discovery as John Wesley's boiled egg-shells for ruptured children.

In this country, in the reign of Charles II., Dr. Jonathan Goddard obtained £5,000. for disclosing his secret for making a medicine, called "*Guttæ Anglicanæ*." And in 1739, the Parliament of England voted £5,000. to Mrs. Stevens for a solvent for stone; notwithstanding which, there have been as many human calculi, since formed by his majesty's liege lithotomical subjects, as would macadamize one side of Lincoln's Inn Fields.

The celebrated David Hartley was very instrumental in procuring this grant to Joanna Stevens. He obtained also a private subscription to the amount of £1356., published one hundred and fifty-five *successful* cases, and, by way of climax to the whole, after eating *two hundred pounds weight* of soap! David himself died of the stone.

Mead's specific for hydrophobia, "*Pulvis Antylisus*," sanctioned by the authority of great names and great learning, shared a similar fate: though Mead, the learned and illustrious Mead, in his recommendation of it, declared, that, in the experience of thirty years, upon more than five hundred patients, he never knew it fail of success! In the Gentleman's Magazine, however, an unsuccessful case is related. "W. Jones, a farmer at Milton, (July 20, 1735,) died of hydrophobia, after taking Mead's powder;" but, the failure is apologised for, from the circumstance of his being bit in the *nose*, "*which being so near the principal parts*, might possibly, (*nosological logic truly*,) prevent his being cured by Dr. Mead's remedy, so successful in cases of this dreadful malady."

England has been called the "Paradise of Quacks." Our ancestors were a nostrum-loving race from the king to the cottager, and the history of panaceas and specifics, in the form of elixirs, pills, powders, and waters, would form a large volume of humiliating memorials of the credulity of the public who could swallow them, and the infatuation of the physician who could prescribe them. Who could believe that a philosopher would eat two hundred pounds of soap?—a bishop drink a butt of tar-water? or that, in a course of chemical neutralization, Meyer, should swallow *twelve hundred* pounds weight of crab's eyes!"

In the German *Ephemerides*, the case of a person is described, who had taken too much Elixir of Vitriol, that his keys were rusted in his pocket, by the transudation of the acid through his skin; and another patient is said to have taken Argentum Nitratum, in solution, till he became blue. But all these philosophers, doctors, and divines, sink into insignificance, before Samuel Jessop, who died at the age of 65, in 1817, whose inordinate craving for physic led him to take, in twenty-one years, no less than 226,934 pills, besides 40,000 bottles of mixture; and in the year 1814, when his appetite increased, his consumption of pills was 51,590!!! Truly he must have thought with the prophet, "The Lord hath created medicines out of the earth, and he that is wise will not abhor them."—Eccles. xxxviii. 5, 6.—*Ibid.*

*Inquest Extraordinary.*—The following extraordinary case, connected with Medical Jurisprudence, appeared a few days ago in the public papers. As there is much obscurity attending it, we refrain from offering any comments upon it at present:—

On Thursday, the 4th ultimo, an inquest was held at Dover, before John Shipden, Esq., Mayor and Coroner, on the body of John Weston, one of the packet porters of that place; and as the circumstances attending it have occasioned a great sensation, and been the subject of various conflicting opinions, we shall lay the fullest particulars before our readers. It appears that the deceased, who had been labouring under a rheumatic affection, had obtained a presentation ticket for relief and medical attention at the Dover Dispensary,—an institution chiefly under the care of a Mr. Sankey, surgeon in that town. Bleeding was prescribed; which operation was performed on Tuesday, the 2d of October, by Mr. Sankey's assistant, and the arm bound up as usual. In the course of the night the arm swelled, and became exceedingly uneasy; and the poor fellow (who had formerly lived as servant to a medical man) was heard to express his strong doubts as to the scientific performance of the operation. Becoming worse, the wife fetched Mr. Sankey, who, with his assistant, examined the arm, in private, and continued with the patient some time; he, however, gradually sunk, and expired about four o'clock on Thursday morning.

A Committee of the Subscribers to the Dispensary was convened, and many persons having stated that they should not be satisfied unless an inquest were held, a precept was accordingly issued by the Mayor and Coroner. At the inquest, the surgeon to the Dispensary (Mr. Sankey) and his assistant alone were examined! The substance of Mr. Sankey's evidence was, '*that the artery had not been cut* (as was popularly reported), *but that he had tied it!*' Neither the evidence of the wife nor of any other person was adduced; and the jury returned their verdict—'*Died by the visitation of God.*' This, however, was far from being satisfactory to the public, who perceived an incongruity in the evidence which seemed to have escaped the jury; and an application was made to the Coroner, to have the matter re-investigated; in consequence, he deputed two medical gentlemen from Deal (Dr. M'Arthur and Mr. Hulke,) with another of the profession at Dover (Mr. Norwood,) to examine and report on the body of the deceased.

The report stated, 'the medical inspectors had found that *about one inch of the brachial artery had been removed* from below the vein which had been opened in bleeding, *and that they found two ligatures* above the part of the artery which had been cut away; of course they were unable to decide whether the artery had, or not, been punctured in the operation of bleeding.' On this report, the Coroner *did not see occasion* for another inquest; and the body was buried on the Sunday.

Public feeling, however, was excited still farther, rather than allayed, by the knowledge of the substance of this examination, and rose to such a height, that the Coroner was obliged to yield to the general wish; and after some delay, another inquest was summoned, the body being disinterred, for the inspection of the jury, which was not entirely composed of the same individuals as at first. The medical witnesses were again examined; and Mr. Sankey deposed, that 'he thought, after the man had been bled, *an internal arterial bleeding had occurred*, and he therefore *tied the artery* above, and adopted the usual means to prevent inflammation; that after death he had ascertained that the artery was not cut; that with regard to the portion of the artery which was missing, he had directed it to be 'cut away, and slit up longitudinally, to ascertain if disease existed.' *It was not produced in court.*

On calling for the evidence of the wife, it was stated in court that she was in strong fits, and accordingly her attendance was dispensed with; but the next day this was discovered *not to have been the case,* as the poor woman was actually waiting in expectation of being summoned, as was also the lady who had recommended Weston to the Dispensary, to support her in court. She states *that she was not sent for.* How this arose, is not precisely understood.

The evidence here terminating, the jury retired, but could not agree in opinion, several refusing positively to sign the verdict determined on by the rest. However, on the principle of the opinion of the majority constituting a legal verdict, it was again recorded—'*Died by the visitation of God!*'

It is needlsss to say, that not much greater satisfaction exists at the present moment than before the second inquest: and in justification of those public-spirited individuals who exerted themselves so laudably in endeavouring to throw a light on this mysterious affair, we have given such particulars of the proceedings as have transpired. *Reporters were not allowed by the Coroner!* It is rumored that a representation of the whole business will be laid before the Secretary for the Home Department.—(*Newspapers and Med. Repository.*)

